# Siblicide between fertilized and unfertilized ovaries within the maize ear

**DOI:** 10.1038/s42003-025-07784-8

**Published:** 2025-03-31

**Authors:** Cheng Huang, Zhi-Wei Wang, Yi-Hsuan Lin, Xiao-Gui Liang, Hui-Min Chen, Bo Hong, Xian-Min Chen, Ya-Ning Zhou, Zhen-Yuan Chen, Shuai Dong, Xin Wang, Si Shen, Shun-Li Zhou

**Affiliations:** 1 College of Agronomy and Biotechnology, China Agricultural University, 100193 Beijing, China; 2 Research Center on Ecological Science, Jiangxi Agricultural University, Nanchang, China; 3 State Key Laboratory of Maize Bio-breeding, China Agricultural University, 100193 Beijing, China; 4 Innovation Center of Agricultural Technology for Lowland Plain of Hebei Province, Wuqiao, China

**Keywords:** Plant physiology, Ecophysiology

## Abstract

Evolutionarily, plants overproduce ovaries but selectively eliminate those inferiors to ensure competitive offspring to set. This sibling rivalry, reducing grain number, is detrimental agronomically. However, the interaction between early-fertilized and unfertilized ovaries in sequentially-pollinated panicles is unclear. Here, we fertilized the ovaries on half rows of maize ear (HP) while keeping the rest unfertilized to investigate their interaction. HP reduced the growth of unfertilized ovaries while promoting fertilized ovary (grain) development. ^13^C-isotope labeling of grains led to isotope signal detected in the unlabeled ovaries, validating their interactions. Transcriptionally, HP caused cell wall degradation and senescence of unfertilized ovaries, reducing their viability. These ovaries showed promoted auxin and jasmonic acid levels with activated auxin signaling but suppressed MAPK signaling. Conversely, HP grains activated MAPK signaling, sugar utilization, and cell proliferation. These findings demonstrate that grains suppress ovaries in ear to consolidate sugar utilization advantage for development, potentially through hormone and MAPK signaling.

## Abstract (web summary)

Grains suppress ovaries in ears of maize to strengthen their sugar utilization advantage for development, potentially through hormone and MAPK signaling.

## Introduction

Angiosperm plants overproduce ovules or ovaries but eliminate those inferior or with low survival probability to invest limited resources into more competitive offspring, which is an important evolutionary strategy for reproduction and bloodline inheritance^1^. However, this strategy of surviving the fittest by sacrificing the rest is detrimental agronomically since it reduces grain number, an important factor determining crop yield^2,3^. For most crops, the flowering stage is the critical window period of determining ovary fate and thereby grain number, when these inferior ovary siblings fail in transition into grain and are aborted. The degree of abortion can reach as high as 80% under unfavorable environmental conditions during the window period, severely limiting yield potential^2,4,5^. Interestingly, abortion usually occurs non-randomly, preferentially in specific regions of the panicles bearing a large population of offspring^6–9^. For instance, the apical region of maize ear^3^, the apical and basal regions of wheat spike, and the central region of sunflower capitulum^10^, demonstrating a hierarchy in the survival competition among these grain siblings on a panicle^11^.

The flowering or pollination of florets or ovaries within a spike or ear is usually asynchronous sequential fertilization has been suggested to determine the hierarchy of ovary siblings in competition^11^. On maize ear, silks from the apical region emerge and are pollinated 3 ~ 5 days later than those from the basal region, leading to a higher risk of abortion for the grains in the apical region^3,12^, while reversing this acropetal pattern of pollination via manually delaying pollination to the basal region correspondingly induced abortion of the basal grains but promoted the apical ones to be set, and eliminating the asynchrony of pollination via synchronous pollination of the ear largely alleviated abortion^2,3^. To understand this basis of sequential fertilization-determined hierarchy in competition among siblings, a model of ‘ready-set-growth’ was proposed that pollination and fertilization trigger the development of the grain with elaborated regulations of sugar and hormone metabolisms and their signaling conductions^11,13,14^. From the perspective of assimilate uptake, the players facilitating sugar uptake in ovaries or grains, such as cell wall invertase (CWIN) and sugar transporters, are strongly stimulated by the signals released from pollination and fertilization to initiate the import and utilization of sugars for ovary or grain set^15^. Spatially, genes encoding CWIN and its inhibitor exhibited a dispersed expression in the ovary before fertilization but changed into phloem-specific expression after being fertilized, indicating stimulation of phloem unloading of sugars by fertilization during the ovary-to-fruit transition^16^. Additionally, fertilization-induced synthesis of auxin initiates the division of central cells in the ovule^17^. As such, the sequential pollination or fertilization asynchronously activates the uptake of assimilates among ovary siblings on a panicle, thus establishing an inherently spatiotemporal hierarchy in competition and determining the developmental fate of grain siblings^11^.

Notably, based on our previous studies, we have proposed that these siblings on a panicle may interact with each other, with the early-pollinated florets or ovaries emitting “death signals” to suppress the development of un-pollinated or later-pollinated ones and induce abortion^11^. A recent study has demonstrated that the post-anthesis florets sent some unknown signals to the younger florets on a sunflower capitulum to permit or deny the anthesis of these younger florets^18^. Similarly, on maize ear, these early-pollinated ovaries were implied to suppress the growth of un-pollinated silks through some signals^19^. However, the signals modulating sibling’s interaction within panicle or ear are largely unknown. Previous studies have shed light upon this basis of interaction by demonstrating the translocation of signal molecules across different tissues to perform regulation roles. For instance, auxin produced in the endosperm upon fertilization is translocated into the receptacle to stimulate fruit development in strawberry^20^, and mobile auxin is involved in modulating the proliferation of inflorescence in other species^21–23^. Other hormones, such as cytokinin, abscisic acid, and ethylene, may also be involved in the ovary or grain sibling interactions^2,24–27^. Besides hormones, sugars, which are highly movable in plants, have been demonstrated to be translocated across different tissues and play regulation roles in the development of inflorescence and ovary^28,29^. Nevertheless, up to date, whether and how these early-fertilized ovaries interact with their siblings on a panicle are still not well understood.

In this study, we selected maize, one of the most important cereals, bearing a large number of ovaries on the ear, as a typical material to investigate the interactions between the early-fertilized ovaries with the unfertilized ovaries. Yet, the sequential pollination nature of the ear makes it challenging to distinguish fertilized ovaries and unfertilized ovaries at an early stage, and inherently, the spatial hierarchy of the ear is coupled with the pollination sequence that may interfere with the investigation. To address these issues and better serve the purpose, this study applied an innovative approach of controlled pollination to manually pollinate half rows on one side of the ear while keeping the other side unpollinated, with complete pollination and non-pollination of the whole ear as the positive and negative controls, respectively, which allows for easy distinction of these fertilized ovaries with unfertilized ovaries at an early stage while eliminating the interference from the spatial hierarchy of different ear region. Pollination treatments assisted with in vitro cultivation of ear section, ^13^C-labeling, metabolic analyses, and transcriptome analyses, this study aims to verify the interaction that exists among siblings within the ear and explore the underlying regulation mechanisms. The findings of the current study would be valuable for ecologists and agronomists, providing evidence and a basis for understanding offspring interaction in flowering plants and the regulation of grain fate in crop production.

Here, our findings showed that half-pollination treatment promoted the growth of fertilized ovaries (grains) while suppressing the development of unfertilized ones. ^13^C isotope labeling further revealed that the isotope signal was transferred from labeled grains to unfertilized ovaries, confirming the existence of sibling interaction within the ear. Transcriptional and metabolic analyses suggested that this interaction is mediated by hormone and MAPK signaling. Specifically, unfertilized ovaries exhibited elevated 3-indoleacetic acid (IAA) and methyl jasmonate (MeJA) levels with activated auxin signaling but suppressed MAPK pathways, whereas MAPK signaling, sugar utilization, and cell proliferation were enhanced in developing grains.

## Results

### Phenotypic responses of ovary and grain to controlled pollination

**Fig. 1:**
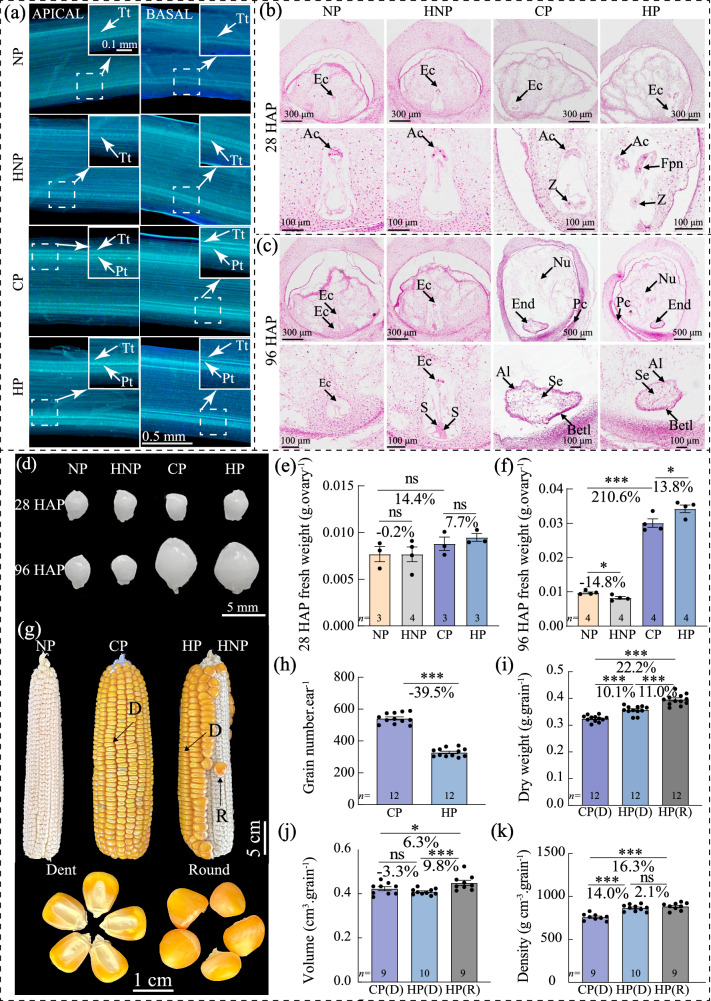
Phenotypes of pollen tubes and ovaries after pollination and at maturity validate the effect of pollination treatments. **a** At 28 HAP, aniline blue-stained pollen tubes were observed in the transmitting tract of silks in CP and HP (both apical region outside of the bract and basal region adjacent to the ovary), while absent in NP and HNP, validating the manual pollination treatments and entry of pollen tubes into ovaries for fertilization. **b** At 28 HAP, in CP and HP, zygote and fertilized polar nuclei with enlarged embryo sac were observed, note that the nucellus was degrading. In NP and HNP, the embryo sac and nucellus remained intact. **c** At 96 HAP, endosperms underwent cellularization in both CP and HP, whilst embryo sac (antipodal cells and synergid) in NP and HNP was intact and still. **d** The grains in CP and HP were visually enlarged compared to that in NP and HNP. **e**–**f** Significant differences in ovary/grain fresh weight were observed between NP vs CP, NP vs HNP, and CP vs HP at 96 HAP. **g** At maturity, compared with normal dent grains in CP, some of the grains in HP were with round shape due to lack of physical extrusion from adjacent grains (HNP). **h**–**k** At maturity, approximate 40% ovaries were unfertilized in the HNP treatment, which resulted to a promotion in weight, volume and density of HP grains on the other side of ear. Values are mean with standard error. *n* represents numbers of biologically independent samples. Student’s *t*-test, significant differences are indicated by asterisks, *p < 0.05; **p < 0.01; ***p < 0.001; ns no significance, HAP hours after pollination, NP non-pollinated ovaries, CP completely pollinated ovaries, HP half pollinated ovaries, HNP half non-pollinated ovaries, Tt transmitting tract, Pt pollen tube, Ac antipodal cells, AL aleurone, Betl basal endosperm transfer cell, Pc placento-chalazal, Ec embryo sac, Fpn fertilized polar nuclei, S synergid, Se starchy endosperm, Z zygote, Nu nucellus, End endosperm, D dent, R round.

To validate the effect of controlled pollination, silks, and ovaries were examined with microscopy at 28 and 96 h after pollination (HAP) (Fig. 1a–c). At 28 HAP, pollen tubes were observed across the base of the silks, and zygote and/or fertilized polar nuclei could be observed in the embryo sacs of ovaries in complete pollination (CP) and half pollination (HP), validating the pollination effect and fertilization at 28 HAP (Fig. 1a, b). As negative control, no pollen tube was observed in the silks with non-pollination (NP) or half non-pollination (HNP) treatments (Fig. 1a). At 96 HAP, the fertilized ovaries (grains) in CP and HP were observed to be enlarged with cellularized endosperm (Fig. 1c). In contrast, the unfertilized ovaries with NP and HNP treatments were not enlarged, and the nucellus was shrunken (Fig. 1c).

Since this study focused on sibling’s interaction at the ovary-to-grain transition stage, a short but crucial window period^11^, the subsequent investigation was at 28 and 96 HAP, representing fertilization and cellularization for endosperm development, respectively. At 28 and 96 HAP, the weight of grains (CP) was 14.4% and 210.6% higher than the unfertilized ovaries (NP), respectively (Fig. 1d–f, Supplementary Data 1). The weight of ovaries in HNP was reduced by 0.2% and 14.8% by early-fertilization of their siblings within the ear at 28 and 96 HAP, respectively, compared with NP; whereas, the weight of grains in HP was promoted by 7.7–13.8% at 28 and 96 HAP, respectively, when their ovary siblings were not fertilized (Fig. 1e, f, Supplementary Data 1), revealing the influence between the ovary and grain siblings within ear have emerged since early development stage. At maturity, grain number per ear with HP treatment was reduced by 39.5% compared with CP (Fig. 1g, h, Supplementary Data 1). Notably, grains in HP comprised both dent grains (normal type as in CP) and round grains presented in the boundary of HP and HNP (Fig. 1g). Notably, the dent and round grains of HP were 10.1% and 22.2% heavier than that of CP, respectively (Fig. 1i, Supplementary Data 1). Specifically, in HP treatment, the volume of round grains was promoted by 6.3%, and the densities of dent and round grains were promoted by 14% and 16.3%, respectively, compared with CP (Fig. 1j, k, Supplementary Data 1). Notably, compared with CP grains, HP grains had similar volume but higher density and dry weight, indicating an incomplete filling of CP grain and an enhanced accumulation of dry matter by HP, which is consistent with the observation of sparser starch granules in maize endosperm under CP^30^.

### ^13^C signals are translocated among grain and ovary siblings via the cob

**Fig. 2:**
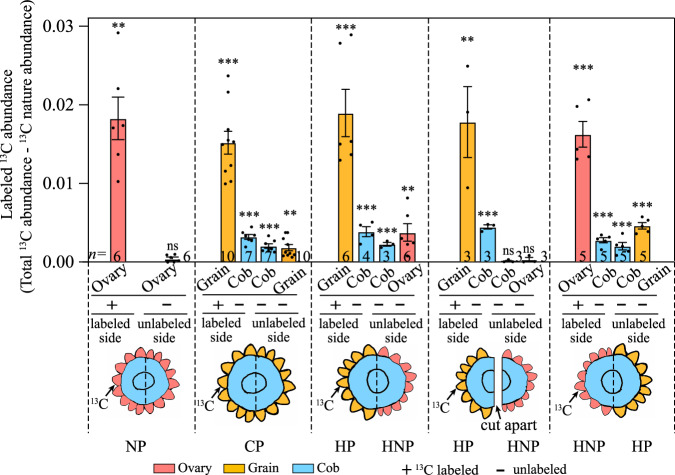
In vitro cultivation of ear section with ^13^C-isotope labeling one side of the ovaries/grains at 96 HAP. When all ovaries within the ear were unfertilized, labeling one side with ¹³C isotope did not show any ¹³C signal on the opposite side of the ear. However, when there were fertilized ovaries, labeling either the fertilized ovaries (grains) or unfertilized ovaries resulted in a significantly higher ¹³C isotope abundance detected on both the cob and the opposite side grains or ovaries, compared to controls. No ¹³C signal appeared on the unlabeled side of the cob and grains when the ear was cut apart, which indicates that fertilization likely initiates the transport of ¹³C molecules between ovary and grain siblings through the cob. Values are means with standard errors, calculated from the total isotopic ^13^C abundance minus the ^13^C natural abundance. *n* represents numbers of biologically independent samples. Student’s *t*-test, Significance is determined by comparison with controls and is indicated by asterisks, *p < 0.05; **p < 0.01; ***p < 0.001; ns no significance, NP non-pollinated ovaries, CP completely pollinated ovaries, HP half pollinated ovaries, HNP half non-pollinated ovaries.

To test whether any signal molecule may translocate between ovary and grain siblings within the ear, the fertilized ovaries (grains) or unfertilized ovaries from one side of the ear section was labeled with ^13^C isotope, a basic and common element constituting signal molecules such as sugars and hormones. In those ovaries or grains with ^13^C labeling (indicated by +), the labeled ^13^C abundance was significantly higher compared to the natural ^13^C abundance (indicated by *), validating the success of isotope labeling (Fig. 2, Supplementary Data 1). Labeling of NP ovaries from one side of the ear did not lead to any significant increase in labeled ^13^C abundance in these unlabeled ovaries on the other side (Fig. 2, Supplementary Data 1). Notably, ^13^C labeling of fertilized grains led to significant increase in labeled ^13^C abundance in both fertilized grains or unfertilized ovaries on the other side of the ear, with ^13^C abundance significantly increased in the cob, in addition, labeling the HNP ovaries also led to ^13^C abundance increased in the HP grains (Fig. 2, Supplementary Data 1), suggesting fertilization-initiated movement of ^13^C-labeled molecules between grain or ovary siblings within ear. Importantly, cutting the ear section apart prevented the ^13^C isotope translocated from the labeled side to the unlabeled side of the ear section (Fig. 2, Supplementary Data 1), validating that the ^13^C-labeled molecules were translocated through the cob tissue. As the ovary-to-grain transition stage is characterized by excessive C assimilates temporarily stored in the maternal cob that is unnecessary and biologically meaningless to translocate C assimilates among grain or ovary siblings^11,31^, the movement of ^13^C isotope was more likely due to, or at least imply that some C-based signaling molecules, rather than C assimilates, were transported among ovary and grain siblings via the cob, and this process was triggered by fertilization of some of the grains on the ear.

### Global responses of ovaries or grains to sequential pollination revealed by transcriptomes

To explore the initial regulations of siblings’ interaction at early development stage rather than the long-term consequences at later development stage, we conducted RNA sequencing on the ovaries and grains with different pollination treatments at 28 and 96 HAP, representing the time points when fertilization just accomplished and the endosperm cells proliferated to build sink capacity, respectively. Overall, there were 35515 genes expressed (Transcripts per million (TPM) > 1) in at least one sample, based on their expression patterns, the NP and HNP were clustered together, as were the CP and HP (Supplementary Fig. 1, Supplementary Data 2). The Spearman’s correlation coefficients of global gene expression between biological replicates were mostly between 0.90 to 0.95 with few exceptions ranging from 0.83 to 0.9 (Supplementary Figs. 2, 3), validating the quality of transcriptome.

**Fig. 3:**
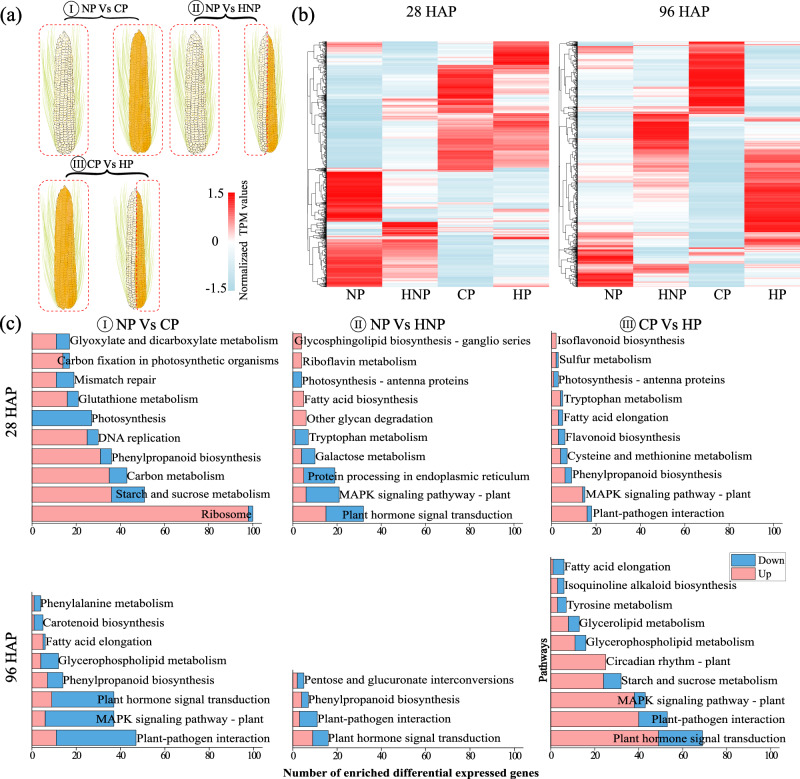
Differentially expressed genes (DEGs) pattern and Kyoto Encyclopedia of Genes and Genomes (KEGG) pathway enrichment reveal transcriptomic interactions among ovary siblings at 28 and 96 HAP. **a** Illustration demonstrated three pair comparisons revealing the impacts of half pollination. **b** Heatmap showed distinct expression patterns of 4636 DEGs in different treatments at 28 and 96 HAP. **c** The top 10 enriched KEGG pathways of DEGs between each pair comparisons at 28 and 96 HAP. TPM transcripts per million, HAP hours after pollination, NP non-pollinated ovaries, CP completely pollinated ovaries, HP half pollinated ovaries, HNP half non-pollinated ovaries.

To serve the purpose of the current study, we applied three pair comparisons, NP vs CP (fertilization effect), NP vs HNP (the influence of early-fertilized ovaries (grains) on unfertilized ovaries within ear), and CP vs HP (the influence of unfertilized ovaries on grains) (Fig. 3a). There were totally 4636 differentially expressed genes (DEGs), with the threshold of | fold change (FC)| ≥2 and *p*-values < 0.05 (Fig. 3b). KEGG analysis highlighted the top 10 enriched metabolism pathways for each comparison (Fig. 3c, Supplementary Data 1). For NP vs CP, the enriched pathways at 28 HAP included ribosome, starch and sucrose metabolism, carbon metabolism, phenylpropanoid biosynthesis and DNA replication, the involved genes were mostly upregulated; at 96 HAP, the enriched pathways included plant-pathogen interaction, Mitogen-activated protein kinase (MAPK) signaling pathway, plant hormone signal transduction (Fig. 3c). For NP vs HNP, the enriched pathways at 28 or 96 HAP included plant hormone signal transduction, MAPK signaling pathway, plant-pathogen interaction (Fig. 3c). For CP vs HP, the enriched pathways overlapped at 28 and 96 HAP also included plant-pathogen interaction, MAPK signaling pathway, phenylpropanoid biosynthesis and plant hormone signal transduction (Fig. 3c). The overlapping pathways among different pair comparisons highlight the signaling regulations in the interactions between grains with unfertilized ovaries within ear. Besides, the numbers of enriched DEGs and pathways decreased and increased over time in NP vs HNP and CP vs HP, respectively (Fig. 3c).

### Sugar contents and trehalose-6-phosphate (T6P) metabolism

**Fig. 4:**
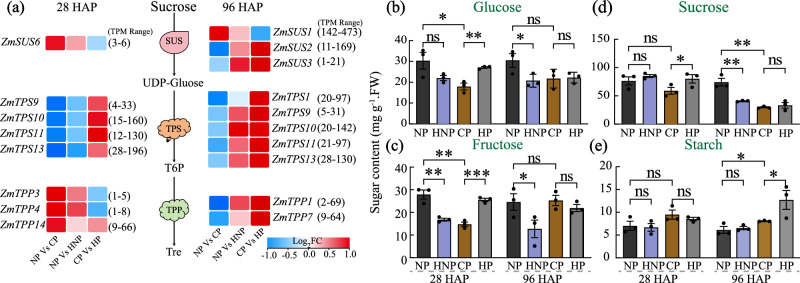
Expression patterns of genes involved in trehalose metabolisms and sugar levels. **a** At 28 HAP, the genes encoding TPS and TPP, which facilitating the synthesis of T6P and converting T6P into trehalose, were suppressed and promoted, respectively, in the HNP ovaries compared with NP ovaries. Whereas, at both 28 and 96 HAP, the *TPS* genes were significantly promoted in the HP grains compared with the CP grains. The absolute values of TPM in brackets represent the range between the minimum and maximum values of the gene in all treatments during that period. The fold change (FC) represents the ratio of the change in TPM value for each comparison. **b**–**e** The levels of endogenous sugars, including glucose, fructose, sucrose, and starch. Note that the levels of hexoses and sucrose were reduced in HNP ovaries at 28 or 96 HAP, but were significantly promoted in HP grains, compared with their controls. Values are mean with standard error. Student’s *t*-test, *n* = 3. Significant differences are indicated by asterisks, *p < 0.05; **p < 0.01; ***p < 0.001; ns no significance, HAP hours after pollination, NP non-pollinated ovaries, CP completely pollinated ovaries, HP half pollinated ovaries, HNP half non-pollinated ovaries, SUS sucrose synthase, TPS trehalose 6-phosphate synthase, TPP trehalose 6-phosphate phosphatase, T6P trehalose 6-phosphate, TPS trehalose.

As sugar signaling plays a key role in regulating development, including ovary-to-grain transition and sugar metabolism that was enriched in response to the pollination treatments^11,32^ (Fig. 3c), we examined the metabolism of T6P (a signaling molecule indicative of sugar availability) and the contents of sugars. At 28 HAP, the HNP ovaries were with lower and higher levels of T6P synthase (TPS)-encoding genes and T6P phosphatase (TPP)-encoding genes, respectively, compared with NP ovaries (Fig. 4a, Supplementary Data 3). Whereas, on the other side of the ear, HP fertilized ovaries (grains) showed promoted *TPS* gene expression but suppressed *TPP* gene expression compared with CP grains (Fig. 4a, Supplementary Data 2). At 96 HAP, the expressions of *TPS* and *TPP* were both promoted by HNP and HP, compared with their controls, NP and CP (Fig. 4a, Supplementary Data 2). These results suggested an interaction between grains and unfertilized ovaries within ear may involve T6P metabolism. Specifically, the differential expressions of *TPS* and *TPP* genes indicated that grains might be promoted in sugar availability for grain development, while these unfertilized ovaries are simultaneously suppressed.

Upon fertilization, the contents of glucose, fructose, and sucrose were reduced (except for fructose which was not changed at 96 HAP), whilst starch content was promoted, at 28 and 96 HAP, demonstrated by CP vs NP (Fig. 4b–e, Supplementary Data 1). Notably, fertilization of partial ovaries significantly suppressed the contents of glucose and fructose (at both 28 and 96 HAP) and sucrose (at 96 HAP) in these unfertilized ovaries within the ear, revealed by NP vs HNP (Fig. 4b–e, Supplementary Data 1). On the other hand, non-pollination to partial ovaries significantly promoted the contents of glucose, fructose, sucrose (28 HAP), and starch (96 HAP) in these grains within the ear, compared by CP with HP (Fig. 4b–e, Supplementary Data 1). The results indicated that the status of fertilization of ovaries influences sugar availability in these unfertilized ovaries on the same ear.

### In-depth analyses of hormone and MAPK signaling pathways

**Fig. 5:**
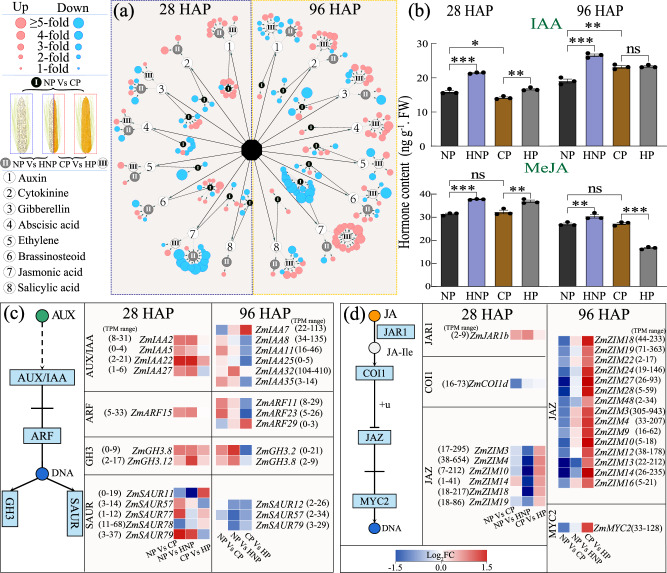
Expression profiles of genes involved in hormone signal transduction and endogenous levels of auxin and JA. **a** Network analysis showed overall responses of hormone signal transduction at 28 and 96 HAP. The signaling pathways of auxin (1) and JA (7) exhibited dramatic changes, where auxin signaling was promoted in the HNP ovaries, and JA signaling was suppressed in the HNP ovaries but promoted in the HP grains. **b** Endogenous levels of IAA and MeJA were consistently promoted in the HNP ovaries at both 28 and 96 HAP, and they were also promoted in the HP grains at 28 HAP but showed inconsistency at 96 HAP. **c**, **d** Genes involved in signal transduction of auxin and JA at 28 and 96 HAP. Players facilitating auxin signaling were mostly promoted in the HNP ovaries, whereas those facilitating JA signaling were mostly suppressed in the HNP ovaries but promoted in the HP grains. The fold change (FC) represents the ratio of the change in TPM value for each comparison. The absolute values of TPM in brackets represent the range between the minimum and maximum values of the gene in all treatments during that period. Values are mean with standard error. Student’s *t*-test, *n* = 3. Significant differences are indicated by asterisks, *p < 0.05; **p < 0.01; ***p < 0.001; ns no significance, HAP hours after pollination, NP non-pollinated ovaries, CP completely pollinated ovaries, HP half pollinated ovaries, HNP half non-pollinated ovaries, IAA Aux/IAA-transcription factor, ARF Auxin response factor, GH3 gretchen hagen 3, SAUR small auxin up RNA, JAR Jasmonic acid-amido synthetase, COI coronatine insensitive, ZIM Zinc-finger protein expressed in inflorescence meristem, MYC Myelocytomatosis, JAZ jasmonate ZIM-domain protein.

As plant hormone signal pathway was enriched and overlapped in different comparisons (Fig. 3c), a global analysis of all hormone signal transduction pathways was conducted (Fig. 5a). As the network analysis shows the numbers and FC of the differentially expressed genes (DEGs) involved in hormone signaling, the pathways of auxin and jasmonic acid (JA) signaling were highlighted to show dramatic DEGs number and FC changes in response to pollination treatment (Fig. 5a). Among them, the players involved in auxin and JA signaling pathways were mostly promoted and suppressed, respectively, by HNP compared with NP at 28 HAP, and JA signaling was promoted in HP compared with CP at 96 HAP (Fig. 5a). Whereas few numbers of players involved in other hormone signaling (brassinolide (BR), gibberellin (GA), cytokinins (CTK), salicylic acid (SA), ethylene (ETH) or abscisic acid (ABA)) were identified to show significant responses among the three comparisons, and mostly had not been functionally characterized (Supplementary Fig. 4a, Supplementary Data 3). The contents change of these hormones only occurred at one stage in NP vs HNP or CP vs HP, except for BR in NP vs HNP and ABA in CP vs HP (Supplementary Fig. 4b, Supplementary Data 1). At 28 and 96 HAP, the levels of IAA and MeJA were significantly promoted in both HNP and HP compared to NP and CP, respectively, except for IAA which was not changed, and MeJA was reduced in HP vs CP at 96 HAP (Fig. 5b, Supplementary Data 1).

The players involved in auxin signaling, *ZmIAAs* and *ZmARFs*, were promoted in the fertilized ovaries (grains) in CP compared to the unfertilized ovaries in NP (Fig. 5c, Supplementary Data 3). Notably, these genes were largely promoted in the unfertilized ovaries in HNP compared with that in the NP at 28 and 96 HAP but were mostly suppressed in the grains in the HP than that in CP (Fig. 5c, Supplementary Data 3). In JA signaling pathway, most of the genes were suppressed by CP and HNP, but promoted by HP, compared with NP and CP, at 28 and 96 HAP, respectively (Fig. 5d, Supplementary Data 3).

**Fig. 6:**
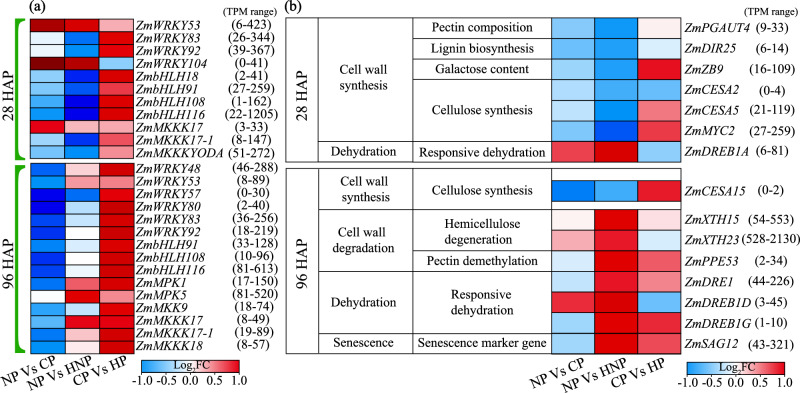
Responses of MAPK signaling and genes relevant to ovary/grain viability at 28 and 96 HAP. **a** Gene expression patterns of DEGs involved in MAPK signal transduction in the three comparisons at 28 and 96 HAP. These genes and transcriptional factors involved in MAPK signaling were largely reduced in the HNP ovaries but largely promoted in the HP grains. **b** DEGs associated with cell wall biosynthesis, degradation, and cell dehydration showed altered expression in the NP vs HNP comparison at 28 and 96 HAP. It was notable that genes facilitating synthesis and degradation of cell wall components were suppressed and promoted, respectively, in the HNP ovaries, accompanied with promoted expressions of dehydration responsive genes and senescence marker gene. The absolute values of TPM in brackets represent the range between the minimum and maximum values of the gene in all treatments during that period. The fold change (FC) represents the ratio of the change in TPM value for each comparison. HAP hours after pollination, NP non-pollinated ovaries, CP completely pollinated ovaries, HP half pollinated ovaries, HNP half non-pollinated ovaries, PGAUT probable galacturonosyltransferase, DIR dirigent protein, ZB zebra crossbands, CESA cellulose synthase, MYC myc transcription factor, XTH xyloglucan endo-transglycosylase/hydrolase, DRE senescence/dehydration-associated protein-related, DREB dehydration-responsive element-binding protein, WRKY WRKY-domain transcription factor, bHLH basic helix-loop-helix (bHLH) transcription factor, MPK mitogen-activated protein kinase, MKK mitogen-activated protein kinase kinase, MKKK mitogen-activated protein kinase kinase kinase.

MAPK modules play key roles in regulation through phosphorylation of downstream signaling targets, including other kinases, enzymes, and transcriptional factors, and have crosstalk between hormone transport and signaling^33^. Here, most of the involved *ZmMAPKs* and their interacting transcription factors (*WRKY* and *bHLH* families) were suppressed in the CP grains compared with the NP ovaries but significantly promoted in the HP grains compared with the CP grains, at 28 and 96 HAP (Fig. 6a, Supplementary Fig. 5, Supplementary Data 3). However, in the HNP ovaries, the *ZmMAPKs* were suppressed at 28 HAP, including *ZmMKK17-1, 17-3, 18-1* and *YODA*, but promoted at 96 HAP, including *ZmMPK1, 5, ZmMKKK17, 17-1, 18*, compared with the NP ovaries (Fig. 6a, Supplementary Fig. 5, Supplementary Data 3).

### Accelerated dehydration and cell integrity alters in HNP ovaries

As the HNP ovaries showed decreased weight compared to the NP ovaries, and the HP grains exhibited increased weight compared to the CP grains, since 28 HAP (Fig. 1e, f), we further investigated the influences on ovaries/grains in response to sibling interaction. Surprisingly, we found that these genes involved in the synthesis of components of the cell wall, including *ZmPGAUT4* for pectin, *ZmDIR25* for lignin, *ZmZB9* for galactose, and *ZmCESA2,5* and *ZmMYC2* for cellulose, were significantly down-regulated, whilst genes involved in cell wall degradation, *ZmXTH15, 23, ZmPPE53*, and dehydration-responsive genes, *ZmDREB1A*, *1D, 1G, ZmDRE1*, were significantly promoted, in the HNP ovaries compared with the NP ovaries, at 28 or 96 HAP (Fig. 6b, Supplementary Data 3). Furthermore, the marker gene of aging, *ZmSAG12*, exhibited a notable up-regulation in the HNP ovaries compared with NP ovaries (Fig. 6b, Supplementary Data 3). These transcriptional responses revealed that the HNP ovaries were inferior in the maintenance of cell integrity and viability but tended toward dehydration and senescence. On the contrary, in the HP grains, most of these genes facilitating cell wall synthesis were promoted, incorporated with significantly promoted expressions of four cell cycle-related genes, compared with CP grains, at 96 HAP (Fig. 6b, Supplementary Fig. 6, Supplementary Data 1), corresponding to the process of endosperm cellularization for grain set during this period (Fig. 1c). These results demonstrated the potential influences on the very early stage of ovary-to-grain transition (within 96 HAP) in response to siblings’ interactions.

## Discussion

Sequential flowering or fertilization of crops such as maize, wheat, and rice are relevant to the hierarchy of ovary/grain siblings within spike or ear, which is closely related to grain number determination and yield potential^3,9,34^. Based on evidence such as phenotypes or basic growth dynamics, it was deduced that these early-fertilized ovaries (grains) may negatively affect the development of unfertilized ovaries or later-fertilized ovaries either through interceptions or competitions of nutrients or through direct interactions with unknown signals^3,5,11,19,35^. To this end, we employed manual pollination to precisely control the fertilization status of ovary siblings within maize ear. With approaches including isotope labeling and RNA-seq, we provide evidence supporting a direct interaction between grains and unfertilized ovaries within the ear via the cob, and propose possible regulation mechanisms through hormone and MAPK signaling. These findings improve the understanding of ovary siblings’ interactions and grain number formation in crops with sequential flowering or pollination of the spike or ear.

### Sequential fertilization drives interactions between grains and unfertilized ovaries within the ear, resulting in suppression of unfertilized ovaries

Conserved in different crops, removal of the superior grains or florets promoted the development of those inferior ones that were otherwise aborted, suggesting a sibling rivalry within spike or ear^3,11,36^. Recently, it was proposed that selective abortion, or failure in the transition to grains, of these inferior ovaries, could result from ‘siblicide,’ with early-developed grains potentially directly or indirectly suppressing the later-developed ones through death signals^11^. Even though, siblings’ interaction remains poorly understood, with several technical issues limiting the investigation. These mainly include (a) in situ distinguishment of early-fertilized ovaries and unfertilized ovaries within a population of inflorescence with naturally sequential pollination, and (b) detecting the signals facilitating or subsequent responses to the interaction at physiological and molecular levels. To this end, this study used approaches of isotope labeling and comparative transcriptome coupled with innovative pollination treatments (Figs. 1g, 2, 3, Supplementary Fig. 7), and hence provided evidence supporting the existence of interactions between ovaries and grains within maize ear.

First, within the ear section, these unlabeled ovaries and cobs could be detected with a significantly increased abundance of ^13^C signal only when their siblings are fertilized (Fig. 2, Supplementary Data 1), suggesting that the siblings’ interaction is conditioned by sequential pollination or fertilization and supporting the possibility of signal molecule translocated within the ear by the cob. Second, it was noted that, just at 96 HAP, the unfertilized ovaries and grains exhibited significantly reduced and promoted weight, respectively (Fig. 1e, f, Supplementary Data 1). As the time point is known with the maximum availability of assimilates (strong photosynthesis and abundant assimilate temporarily stored in stem or cob) but minimum consumption of assimilates by grains compared with the later filling stage^14^, the phenotypical response reveals an active regulation by signals rather than a passive competition of maternal assimilate availability. Third, within 28 and 96 HAP, the unfertilized ovaries in HNP, with some of their siblings being fertilized, showed distinct gene expression patterns versus the unfertilized ovaries in NP that no sibling was fertilized (Fig. 3c, Supplementary Data 1), the transcriptional responses of unfertilized ovaries required grain-derived signals. This is supported by the enrichment of DEGs in pathways related to hormone and MAPK signal (Fig. 3c).

The nature of sequential flowering or pollination in cereals and other agronomically important crops profoundly affects grain number within the narrow time window period of flowering^3,11,18,19,36^, the interaction between unfertilized ovaries and grains within the inflorescence (e.g. spike and ear) is importance for grain number determination and yield potential that deserves further investigation. On the other hand, synchronizing pollination within the ear through manual assistance or genetic improvement of ear architecture significantly improved grain number and restored up to 80% of the grains that would otherwise be aborted under environmental stresses^2,37,38^. Our findings, incorporated with this previous progress, provide a new perspective of manipulation of the populations of grain siblings and their interactions within the inflorescence, rather than the individual grains, to improve crop yield potential.

### Hormone and MAPK signaling modulate the interactions between grains and unfertilized ovaries within the ear

**Fig. 7:**
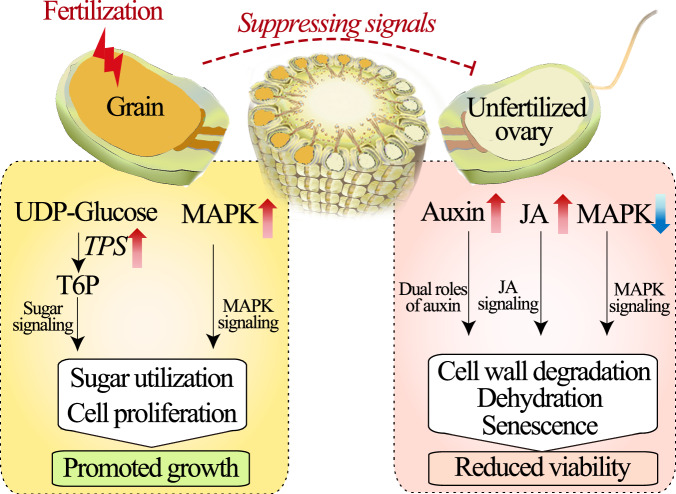
A model depicting the mechanisms by which these early grains suppress these unfertilized ovaries to consolidate their advantage for development. When the ear is asynchronously pollinated, these early grains may emit some suppressing signal molecules that are transmitted into these unfertilized ovaries. Upon receiving the signals, these unfertilized ovaries synthesize excessive levels of auxin and JA with activated auxin signaling transduction, while reducing MAPK signaling. Consequently, the processes of cell wall degradation, dehydration, and senescence are initiated, impeding the viability maintenance of these ovaries. On the contrary, these early grains were promoted with increased sugar availability and activated sugar and MAPK signaling for sugar utilization and cell proliferation. The signal molecule transmitting among grain/ovary siblings has been proposed in current and many recent studies but is still unvalidated. TPS trehalose 6-phosphate synthase, T6P trehalose 6-phosphate, JA jasmonic acid, MAPK mitogen-activated protein kinase.

Taking advantage of comparative transcriptomes, a method capable of detecting early transcriptional responses before the emergence of phenotypic responses, we systematically investigated the responses in NP vs HNP ovaries (Fig. 3c), pair comparisons representing the influences of early developed grains on the unfertilized ovaries within the ear. Surprisingly, since 28 and 96 HAP these unfertilized ovaries in HNP had exhibited largely reduced biosynthesis of cell wall components but promoted degradation of hemicellulose and pectin, incorporated with stimulated expressions of dehydration responsive genes, *ZmDRE1* and *ZmDREBs*, and senescence marker gene, *ZmSAG12* (Fig. 6b, Supplementary Data 3). Considered that (i) cell wall metabolism is closely associated with cell integrity and hence viability of plant tissues including ovary and embryo^39–42^, and (ii) dehydration is a typical symptom of ovary abortion in varies species, the promoted cell wall catabolism and dehydration suggest that these unfertilized ovaries in NP were suppressed and tended toward losing viability (Figs. 6b, 7, Supplementary Data 3).

To explore the regulation basis of siblings’ interaction during the narrow window period of ovary-to-grain transition, hormone, and MAPK signaling pathway, the top two signaling pathways according to KEGG enrichment (Fig. 3c), were further analyzed. We further filtered these hormones by their changes in (i) contents and (ii) gene expressions of signaling pathways in response to siblings’ interaction. For instance, GA and ABA, with no significant difference in content between NP and HNP (at 96 HAP), containing only 10 and 4 DEGs in their signaling pathway, respectively (Supplementary Fig. 4, Supplementary Data 3), were far less responsive than IAA and JA, who showed a significant increase in content and contained more than 20 DEGs in their signaling pathway (Fig. 5, Supplementary Data 1, 3), therefore, we focused on IAA and JA in the subsequent analysis. Specifically, IAA and JA levels were significantly promoted, with auxin transporters (*ZmPIN1* and *4*) being promoted (Fig. 5b, Supplementary Fig. 8, Supplementary Data 1, 3), and their signaling transduction pathways were largely activated and repressed, respectively, in the unfertilized ovaries in HNP (Fig. 5c, d, Supplementary Data 3). Recently, it was demonstrated that excessive auxin levels and signaling inhibited the normal cellularization of the endosperm, ultimately leading to seed abortion^43^. Importantly, auxin-responsive factors involved in auxin signaling, play dual roles in activating post-fertilized fruit growth but inhibit unfertilized fruit sets^44^. Similarly, it was demonstrated that excessive JA inhibits the transition from the mitotic cell cycle to the endoreduplication cycle and induces abortion of unfertilized florets or spikelets across different species^45–48^. In addition, both auxin and JA were reported to play roles in modulating the integrity of the cell wall that relates to ovary vigor and viability^49,50^. These previous findings, incorporated with our findings, support that excessive auxin and JA may be involved in the suppression of unfertilized ovaries in HNP during the narrow window period of sequential fertilization of the ear (Fig. 7).

On the contrary, the development of grains in HP was promoted at an early stage compared with that in CP (Fig. 1f, Supplementary Data 1), characterized by promoted levels of endogenous sugars and significantly increased expression of *TPS* genes that synthesize T6P (Fig. 4, Supplementary Data 1, 3), an important signaling molecule that senses sucrose availability to modulate sugar utilization^51^. At 28 HAP, sugar uptake by the fertilized grains is stimulated to promote the levels of glucose, sucrose, and fructose levels as previously reported^15,16^, and this promotion effect is more dramatic in HP than in CP, may be regulated by some unknown signals that emitted from the maternal tissue. At this time point, these sugars mainly provide energy for embryo and endosperm development and/or act as signaling molecules to promote the early cellulation of endosperm cells before 96 HAP^52^. By 96 HAP, endosperm cells begin rapid proliferation through mitosis^52^, a process that requires sugars to be converted into structural substances, such as cell wall, which is mainly composed of lignin and cellulose——polysaccharides synthesized from hexoses. The rapid utilization of hexoses may narrow the difference in hexose levels between CP and HP. Besides, at this stage, hexoses are also converted into starch to temporarily store in the pericarp^31^, and HP may promote this process to cause a higher starch level. Consistently, genes encoding sugar transporters facilitating sugar uptake by grains^31^, *ZmSUT1*, *ZmSWEET4c*, and *ZmSWEET13*, were simultaneously increased (Supplementary Fig. 9). Remarkably, in the grains of HP, *ZmDOF3*, positively regulates starch accumulation in endosperm development^53^, accompanied by its homologs, *ZmDOF5* and *11*, and *ZmSMR4*, a cell cycle-related gene promoting seed filling^54^, were all significantly promoted (Supplementary Fig. 6, Supplementary Data 3). These results strongly support that reducing sibling population leads to promoted sugar utilization, cell proliferation, and development of the rest grains that are pollinated.

Interestingly, MAPK signaling was largely induced in the HP grains but reduced in the HNP ovaries, respectively (Fig. 6a, Supplementary Fig. 5, Supplementary Data 3). It was demonstrated that the MAPK signaling pathway is broadly involved in adapting to various stress stimuli and regulating gametophyte development^33^. For example, reducing MAPK activity impaired cell wall integrity and reduced the grain number and weight of rice^55–57^. Additionally, our results indicate that both *WRKY* and *bHLH* transcription factors were upregulated in HP grains while downregulated in HNP ovaries, exhibiting contrasting expression profiles in grains and ovaries (Fig. 6a, Supplementary Fig. 5, Supplementary Data 3). Previous studies have shown that TFs from WRKY and bHLH can interact with players involved in MAPK signaling and modulate grain size and weight^58–61^. For instance, overexpression of *OsWRKY53* or *OsWRKY78* increases grain weight, whereas silencing these genes leads to smaller grains, with *OsWRKY53* being positively regulated by MAPK6^59–61^. Furthermore, the *ZmYODA* (*MAPKKK*) activator gene, *opaque11*, encodes an endosperm-specific bHLH; mutants lacking its function exhibit altered carbohydrate metabolism that results in smaller grains^58^. The evidence coupled with current results supports that MAPK signal and its interacting factor WRKY and bHLH may play dual roles in siblings’ interaction, promoting the development of grains while suppressing the viability of unfertilized ovary on the ear (Fig. 7).

### Siblicide among grains and ovaries underlies sibling rivalry and selective abortion within the ear

Selective abortion is prevalent in various crops, where ovaries or florets from a specific region of the ear or panicle are more vulnerable and with a higher frequency of abortion (e.g., the barren tip of maize ear), severely limiting yield potential^11^. Given its detrimental impact on crop production, the regulatory basis of selective abortion has been intensively studied and proposed to be attributed to asynchronous pollination or pollination time gaps within the ear or spike, competition or interception of maternal nutrients, or regulations from hormones such as ABA and ETH^2,3,19,25,62^. Notably, most of the conclusions were drawn by comparing those superior with inferior grains, which allows an exploration of the responses of individual grains rather than any regulatory basis underlying interaction or rivalry among siblings. To this end, an outstanding question was highlighted whether the sibling rivalry-induced selective abortion on an ear or panicle is via “suicide” or “siblicide” or a combination of both^11^. In the suicide theory, selective abortion is autonomously initiated without interference from other grain siblings, whereas the siblicide theory draws a scenery that these unfertilized or later-fertilized ovaries sense death signals emitted by early neighboring grains, or that these earlier grains might indirectly suppress the unfertilized ovaries by maternal tissues^11^.

Here, we propose that the involvement of siblicide in sibling rivalry, supported by evidence including isotope translocation from the grains into unfertilized ovaries via the cob (Fig. 2) and the abundant changes of hormone and MAPK signaling in the unfertilized ovaries (HNP) in response to fertilization of their siblings on an ear (Figs. 5, 6a, Supplementary Data 3). Notably, emerging observations of the siblicide phenomenon in many other species have been reported^18,45^. Our results, incorporated with these findings, imply siblicide may underlie the induction of selective abortion, highlighting its potential conservativeness and significance in sibling rivalry for grain set.

Although the transcriptional responses to interactions within grains and ovaries were investigated by the current study, the signal molecule facilitating siblings’ interaction is unknown (Fig. 7). It is worth noting that auxin, can be translocated from the seed (filial tissue) to the receptacle (maternal tissue) or transported over long distances to play regulation roles^20,63^, was somehow promoted (Fig. 5b, Supplementary Data 1, 3), incorporated with higher expressions of auxin transporter genes, *ZmPIN1* and *ZmPIN4* (Supplementary Fig. 8), in these unfertilized ovaries in HNP. This result implies that auxin is a candidate signaling molecule facilitating sibling rivalry. However, the current evidence could not exclude the possibility of other signaling molecules emitted directly by early grains, as well as the signals processed and transformed by maternal tissues, which may subsequently lead to the inhibition of unfertilized ovaries. Examples include, hormones, or volatile compounds that have been demonstrated to transmit signals across different plants, tissues, or cells^64–66^. For instance, HP treatment promoted both GA content and signaling players, *ZmbHLHs* compared with CP, whereas ABA content showed inconsistent pattern at 28 and 96 HAP (Supplementary Fig. 4, Supplementary Data 1, 3), therefore, the signaling network facilitating the siblings’ interactions may involve multiple players that require further effort.

Collectively, the results of the current study support that sequential pollination of the ear drives interaction (siblicide) between these early-developed grains and unfertilized ovaries, implying the involvement of hormone and MAPK signaling in response to this interaction (Fig. 7). These findings improve the understanding of early development of grains in a perspective of grain colonies rather than individual grain and provide theoretical basis to enhance grain number by synchronizing pollination of ear or alleviating the suppression impact from early-developed grains upon unfertilized ovaries.

## Materials and methods

### Plant materials and management

Maize plants were grown in the field at the Wuqiao Experimental Station of China Agricultural University in Hebei Province, China. Zhengdan 958 (ZD958), a locally typical maize cultivar, was used as material. Maize plants were cultivated at a density of 82,500 plants per hectare, using a basal compound fertilizer (750 kg ha^−1^; N 15%, P_2_O_5_ 15%, K_2_O 15%) and a top-dressing of urea (245 kg ha^−1^; N 46%) applied at the V13 stage. Pesticides were applied to protect the plants from insects and diseases.

### Manual pollination and sampling

The manual pollination was according to Shen et al.^3^ with modifications. Specifically, non-pollination (NP) was set as negative control, two distinct pollination methods were employed: (a) complete pollination (CP), synchronously pollinated to all silks with fresh pollens as positive control; (b) half pollination (HP), in which silks initiated from one side of ear were pollinated with fresh pollens, and the ovaries from the opposite side kept non-pollinated (HNP) (Supplementary Fig. 7a).

Silks and ovaries from the middle region of the ear were sampled at 28 and 96 HAP (Supplementary Fig. 7b). The silks and a portion of the ovaries were used for microscopic observation while another portion of the ovaries were instantly frozen by liquid nitrogen and stored at -80 °C for subsequent RNA sequencing, and determination of sugars and hormones. At maturity, as HP ears contained both dent grains (typical shape in CP) and round grains on the boundary to the non-pollination side (due to lack of physical extrusion from adjacent grains), two shapes of grain were separately investigated to assess the potential weight of single grain with or without adjacent siblings (Supplementary Fig. 7b).

### Microscopic observation of pollen tube and ovary

Pollen tube observation was performed according to Shen et al.^15^ with modifications. Fresh ovaries and silks (from the middle region of the ear) were fixed in vials containing formalin-aceto-alcohol (FAA) solution (70% ethanol: glacial acetic acid: formaldehyde = 1.8:1:1, v/v/v), and the vials were with vacuum infiltration. The silks were treated with 8 M sodium hydroxide (NaOH) for 2 h, followed by 12 h of decolorization in a sodium hypochlorite (NaClO) solution with 4% free alkali. Then, the silks were stained with 0.2% water-soluble aniline blue dye for 6 hours. Observation and imaging were performed through a microscope (Olympus CX41, Japan) with a camera and UV lamp.

Ovaries observation was conducted based on Chen et al.^67^ with minor modifications. The section thickness was 4 µm. Stained sections were examined and imaged using an inverted microscope (Nikon CI-S, Japan) in combination with dedicated software (KF-PRO-120, China).

### Determination of glucose, fructose, sucrose, and starch content

Sugar extraction and determination were conducted following Hendrix and Lin et al.^4,68^ with slight modifications. Briefly, the supernatant and pellet of extraction were separately for measurement of soluble sugar (glucose, fructose, and sucrose) and starch, respectively. For soluble sugar assay, mixed 10 μl soluble sugar extraction with 100 mM HEPES (pH 7.5), 4 mM MgCl_2_, 1 mM NAD^+^, 1.2 mM ATP, 1.5 U hexokinase, 1.2 U glucose-6-phosphate dehydrogenase and water to make a total volume of 300 μl in well plates, incubated for 40 min at room temperature (RT) for glucose assay at a wavelength of 340 nm was read using a Multiskan microplate reader (Thermo Fisher Scientific, USA). Afterward, the reaction mixture was added with 0.17 μl glucose-6-phosphate isomerase (1.18U/μl) and 4.83 μl water to reach a total volume of 305 μl and then incubated for 40 min at RT for fructose assay. Afterward, the reaction mixture was mixed with 1 μl invertase (9U/μl) and 4 μl water (total volume of 310 μl) for 90 min at RT for sucrose assay. Starch was measured by hydrolyzing starch into glucose by amylase and amyloglucosidase and then measured as glucose determination. The contents of sugars were calculated using external standards of glucose, fructose, and sucrose.

### Assay of ovary hormone levels

The phytohormones, including 3-indoleacetic acid (IAA), zeatin riboside (ZR), gibberellin 3 (GA_3_), brassinolide (BR), methyl jasmonate (MeJA), and abscisic acid (ABA), were extracted and quantified using the methods outlined in Yang et al.^69^. A total of 0.5 g of frozen ovaries was placed into a pre-cooled (4 °C) mortar. Then, 10 ml of 80% methanol containing 1 mM butylated hydroxytoluene (Sinopharm Chemical Reagent Co., Ltd, Shanghai, China) as an antioxidant was added, and the mixture was ground into a homogenate. It was allowed to extract at 4 °C for 4 h and then centrifuged at 4000 rpm for 15 min at 4 °C. The supernatant was passed through Chromosep C^18^ columns (C^18^ Sep-Pak Cartridge, Waters Corp, Millford, MA, USA), and prewashed with 10 mL of 100% and 5 mL of 80% methanol. The purified fractions were dried under N_2_. Finally, the volume was adjusted by adding 2 ml of phosphate-buffered saline sample diluent containing Tween-20 and gelatin for measurement. The hormone levels were assessed through enzyme-linked immunosorbent assay (ELISA). The mouse monoclonal antigens and antibodies against IAA, ZR, GA_3_, BR, MeJA, ABA, and immunoglobulin G-horseradish peroxidase (IgG-HRP) used in ELISA were produced at the Phytohormones Research Institute, China Agricultural University, China.

### In vitro ^13^C-isotope labeling and determination

As a stable and quantitatively traceable isotope, ^13^C is applied in plant science research in gaseous form^70–72^. As the gaseous form of ^13^C could not achieve labeling partial of the ovaries while keeping the others unlabeled, we obtained ^13^C-labeled plant tissue (solid-state) according to Gao et al.^70^ with modifications for the next labeling experiment. Briefly, we first introduced 80 ml of gaseous ^13^CO_2_ into the bag and allowed it to permeate for 4 h by leaves. Subsequently, the ^13^C-labeled leaves (~26 g) were ground into homogenates with 60 ml starch solutions (starch: water; w: v; 1:3) to obtain a ^13^C-labeled paste by stirring under ~80 °C. The ^13^C-labeled paste was sterilized at 120 °C and 103 kPa for 20 minutes for sterilization and deactivation (to avoid any ^13^CO_2_ exchange from cell respiration during the following labeling process).

The in vitro culture and medium preparation were according to Zhang et al.^73^ with slight adjustments. Selected ears from each treatment at 96 HAP were cultivated. The culture medium was autoclaved, cooled to RT, and then 80 mg/L kanamycin was added. The middle 5 rings of the ovaries of the ear were taken, with the top 1 ring and the bottom 3 rings of grains removed, leaving only 1 ring of grains on the ear section. For ear blocks of NP, CP, and HP treatments, the ^13^C-labeled paste was applied to the pericarp of ovaries on one side (half-ring of grains), notably, no paste was applied to the cob tissue, and the ear section was placed in the culture medium (Supplementary Fig. 10). We set a negative control by cutting apart the HP ear section from the middle of the cob with ^13^C-labeled paste applied to the pericarp of the fertilized side while leaving the unfertilized side unlabeled. Then the two halves of the cob were cultured in the same medium but physically separated. The labeled paste was exclusively applied to the ovaries without any contact with other tissue or culture medium. Incubate in the dark at 25 ± 1 °C for 5 days. Ears with different pollination treatments but without isotope labeling were used as blank controls.

After incubation, ovaries, grains, and cobs (separated into labeled and unlabeled sides) were separately sampled and thoroughly rinsed with distilled water to remove the ^13^C-labeled paste. All samples were dried at 75 °C to a constant weight (by continually weighting the samples during the drying process) and then ground into powder. The ^13^C abundance was determined using an elemental analyzer (elementar vario—PYRO cube, Germany) coupled with an isotope ratio mass spectrometer (isoprime100, UK). The values of labeled ^13^C abundance were calculated by the ^13^C abundance of the labeled sample minus the natural abundance of ^13^C of the unlabeled sample with the same treatment.

### RNA extraction, library construction, and sequencing

Ovaries at 28 and 96 HAP were used for RNA sequencing. Total RNA was extracted from the samples using the RNA extraction kit (CBL + RN40, Aidlab, China). The concentration and purity of RNA were determined using the NanoDrop 2000 (Thermo Fisher, USA), while RNA integrity was assessed with the Agilent 2100, LabChip GX (PerkinElmer, USA). Qualified samples were used for the RNA-seq library constructed by PCR amplification and purification. The library’s quality was verified using the Qsep-400 method, and sequencing was conducted on the Illumina NovaSeq 6000 platform (Illumina, San Diego, USA).

### Differentially expressed genes (DEGs) screening and bioinformatic analysis

The clean data, obtained after the removal of adapter sequences and low-quality reads from the raw data of each sample, were aligned to the maize reference genome (B73_RefGen_v4, https://www.maizegdb.org/assembly/) using Hisat2. The criteria for defining expressed genes were that the gene expression level (TPM value, Transcripts per million) had to exceed 1 in at least one sample at one time^31,74^. DEGs were identified through the BMKCloud platform (https://www.biocloud.net) using a threshold of |fold-change (FC)| ≥ 2 and *p* < 0.05, based on gene count values. The FKPM (Fragments per kilobase of transcript per million mapped reads) values were converted to TPM values^31^. The normalized expression values of genes with TPM values were used for hierarchical clustering. Metabolic pathway enrichment analysis of DEGs sets for each treatment was performed using the Kyoto Encyclopedia of Genes and Genomes (KEGG) pathway network (https://www.genome.jp/kegg/). Only categories with significant *p*-values (*p* < 0.05) were displayed. The top 10 metabolic pathways with *p*-values < 0.05 were subjected to specific pathway analysis. Gene IDs within the pathways were cross-referenced with the Maize Genome Database (https://maizegdb.org/) and the National Center for Biotechnology Information (https://www.ncbi.nlm.nih.gov/).

### Statistics and reproducibility

The measurements and microscopic analyses described above were conducted with at least three independent biological replicates for statistic and reproducibility (*n*  ≥  3). Statistical analysis was done by Student’s *t*-test using Excel (Microsoft, 2020, US) and GraphPad Prism (GraphPad, 9, US). Bioinformatic analysis was conducted using Tbtools^75^ and R studio (R studio, 4.2.1, US) including packages of ggplot2 and pheatmap. Network analysis of DEGs in metabolic pathways was carried out using Cytoscape (NIGMS, 3.7.2, US). Illustrations were drawn using Adobe Illustrator (Adobe, 2023, US) or Adobe Photoshop (Adobe, 2020, US).

### Reporting summary

Further information on research design is available in the Nature Portfolio Reporting Summary linked to this article.

## Supplementary information

Supplementary Information

Description of Supplementary Files

Supplementary Data 1

Supplementary Data 2

Supplementary Data 3

Reporting Summary

## Data Availability

All relevant data are available in the supplementary information or from the corresponding author upon reasonable request. The numerical source data for the graphs presented in all figures are available in Supplementary Data 1. Accession codes are provided in Supplementary Data 2, while the heatmap data are available in Supplementary Data 3. The RNA-seq raw data was deposited in NCBI’s Sequence Read Archive (PRJNA1224472).
