# Supplementary material for: Siblicide between fertilized and unfertilized ovaries within the maize ear
Source: Commun Biol. 2025 Mar 31;8:528. doi: 10.1038/s42003-025-07784-8 (PMC11958663; doi:10.1038/s42003-025-07784-8)
Supplement: Supplementary file 1 — Supplementary Information [file 42003_2025_7784_MOESM1_ESM.pdf]

Supplementary Figure 1

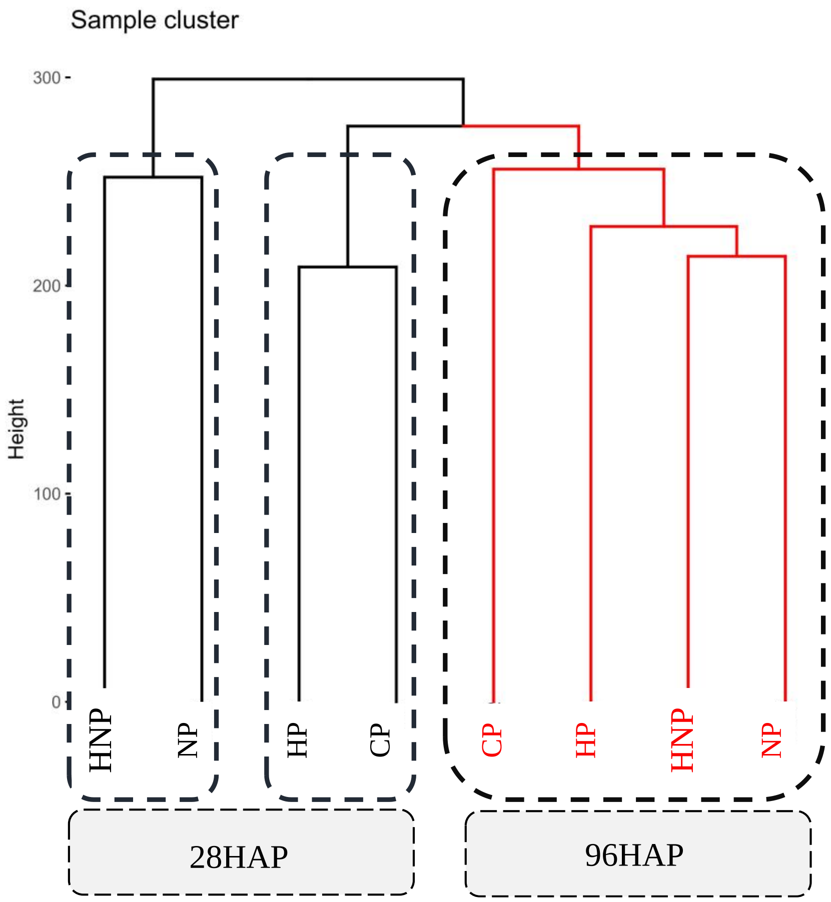

Supplementary Figure 1 Clustering analysis of expressed genes.
Cluster analysis of expressed genes in the samples of three treatments at 28 and 96 HAP. HAP, hours after pollination; NP, non-pollinated ovaries; CP, completely pollinated ovaries; HP, half pollinated ovaries; HNP, half non-pollinated ovaries.

Supplementary Figure 2

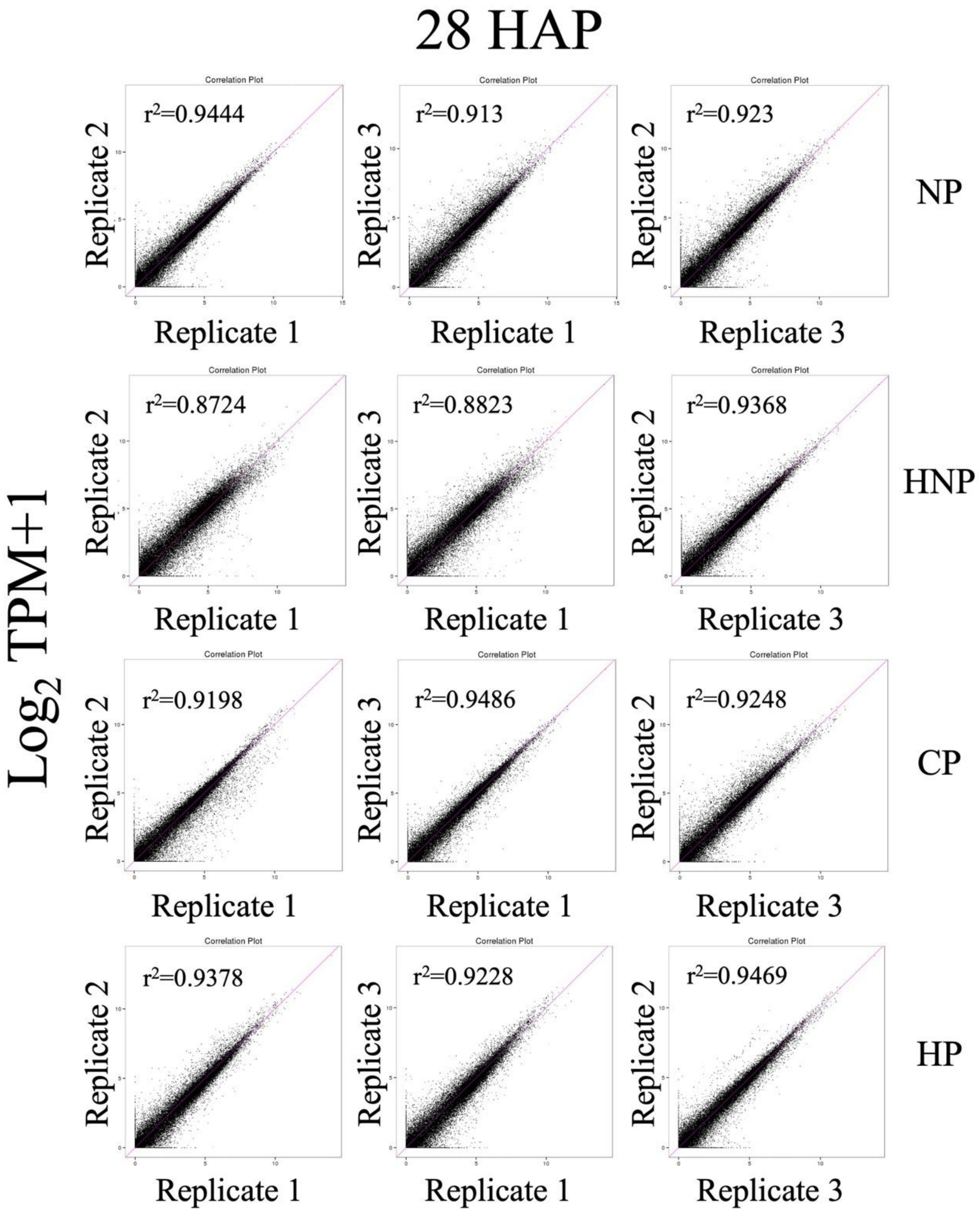

Supplementary Figure 2 Correlations of gene expression patterns (at 28 HAP) among biological replicates validate the reliability of sampling and transcriptome for analyses. Three biological replicates were collected for each treatment, and RNA-seq libraries were independently constructed. Expression levels (TPM) for each replicate were calculated, and pairwise comparisons were made. The normalized data of $\log_2$ (TPM value + 1) was used to calculate the correlation coefficient (r^2 ranges from 0.8724 to 0.9486).

HAP, hours after pollination; NP, non-pollinated ovaries; CP, completely pollinated ovaries; HP, half pollinated ovaries; HNP, half non-pollinated ovaries.

Supplementary Figure 3

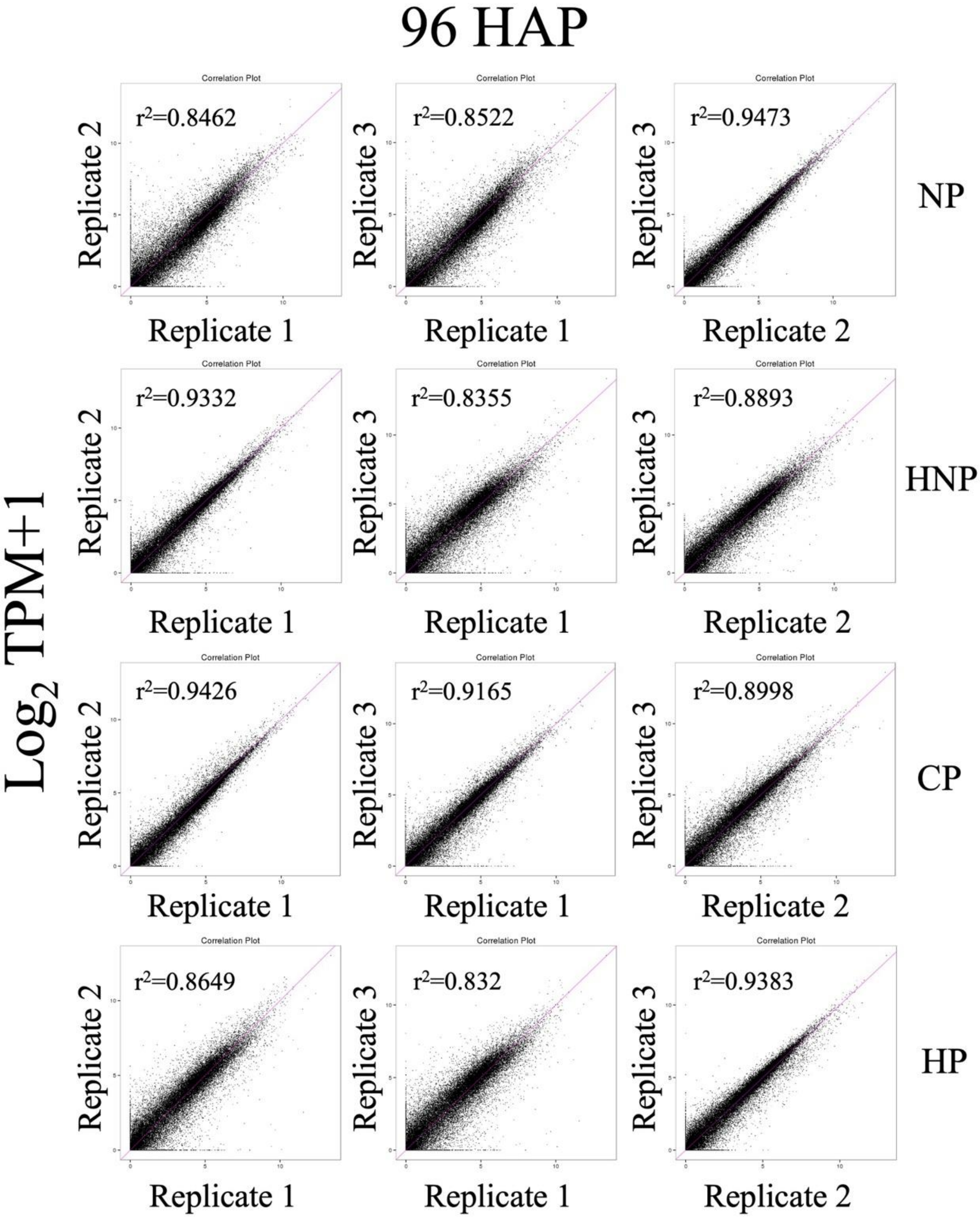

Supplementary Figure 3 Correlations of gene expression patterns (at 96 HAP) among biological replicates validate the reliability of sampling and transcriptome for analyses. Three biological replicates were collected for each treatment, and RNA-seq libraries were independently constructed. Expression levels (TPM) of each independent replicate were calculated, and pairwise comparisons were performed. The normalized data of $\log_2$ (TPM value + 1) were used to calculate the correlation coefficient (r^2 ranges from 0.8320 to 0.9473).

HAP, hours after pollination; NP, non-pollinated ovaries; CP, completely pollinated ovaries; HP, half pollinated ovaries; HNP, half non-pollinated ovaries.

Supplementary Figure 4

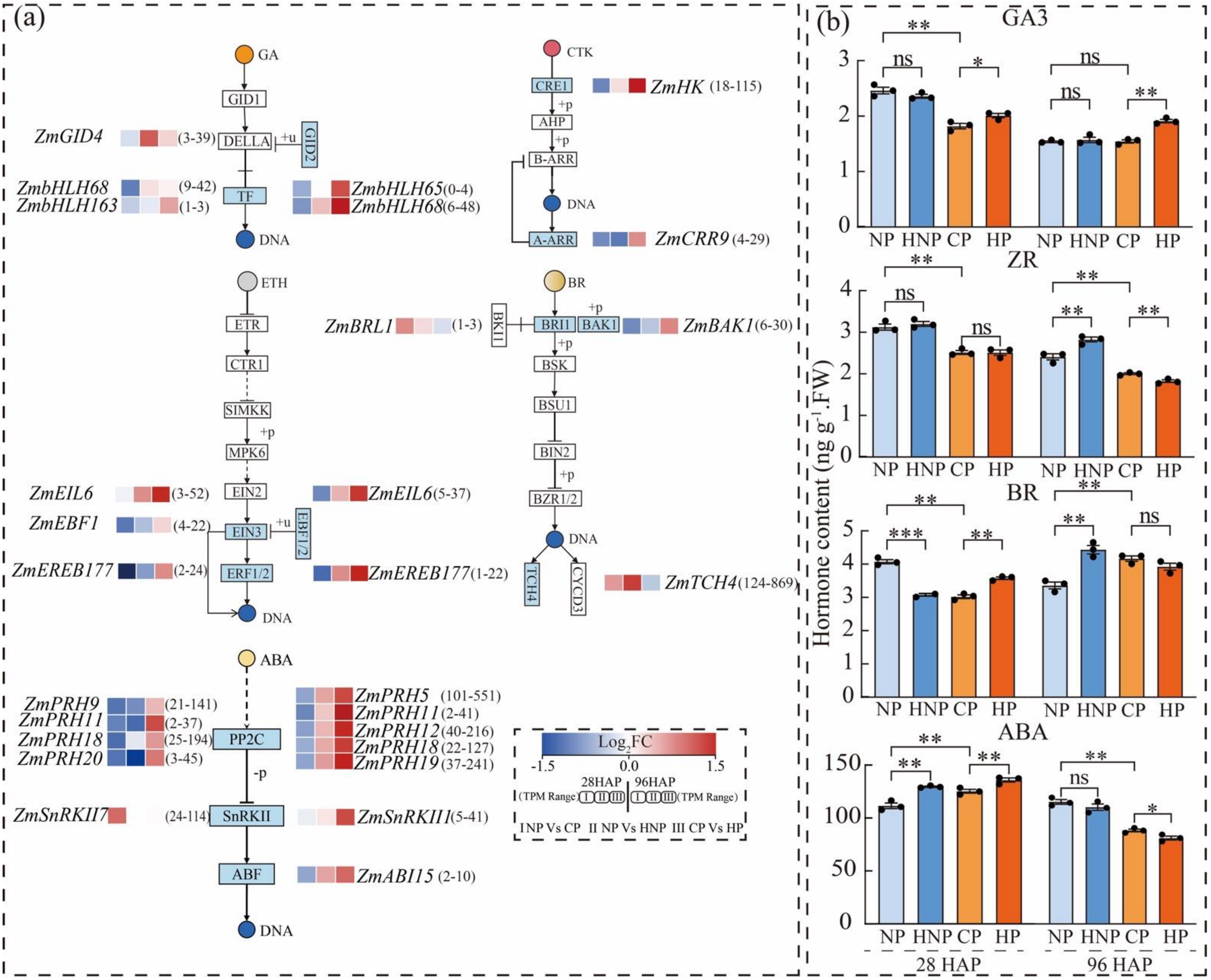

Supplementary Figure 4 Gene expression profile of signal transduction and hormone contents.

(a) Expression pattern of DEGs involved in gibberellin (GA), cytokinin (CTK), ethylene (ETH), brassinosteroid (BR) and abscisic acid (ABA) signaling pathways.

(b) Evaluation of the contents of gibberellin 3 (GA3), zeatin-riboside (ZR), brassinosteroid (BR) and abscisic acid (ABA) in maize ovaries among the three treatments.

The absolute values of TPM in brackets represent the range between the minimum and maximum values of the gene in all treatments during that period. The fold change (FC) represents the ratio of the change in TPM value for each comparison. Values are mean with standard error. Student's *t*-test, *n* =3. Significant differences are indicated by asterisks.*$p<0.05$; **$p<0.01$; ***$p<0.001$; ns, no significance. HAP, hours after pollination; NP, non-pollinated ovaries; CP, completely pollinated ovaries; HP, half pollinated ovaries; HNP, half non-pollinated ovaries; GID, gibberellin-insensitive dwarf protein homolog; bHLH, basic helix-loop-helix transcription factor; HK, histidine kinase; CRR, cytokinin response regulator; EIL, ethylene insensitive-like; EIF, EIN3-binding F-box protein; EREB, AP2-EREBP-transcription factor; BRL, brassinosteroid insensitive1-like receptor kinase; BAK, brassinosteroid insensitive1-associated receptor kinase; TCH, xyloglucan endo-transglycosylase/hydrolase; PRH, protein phosphatase homolog; SnRKII, SnRK2 serine threonine protein kinase; ABI, ABI3-VP1-transcription factor;

Supplementary Figure 5

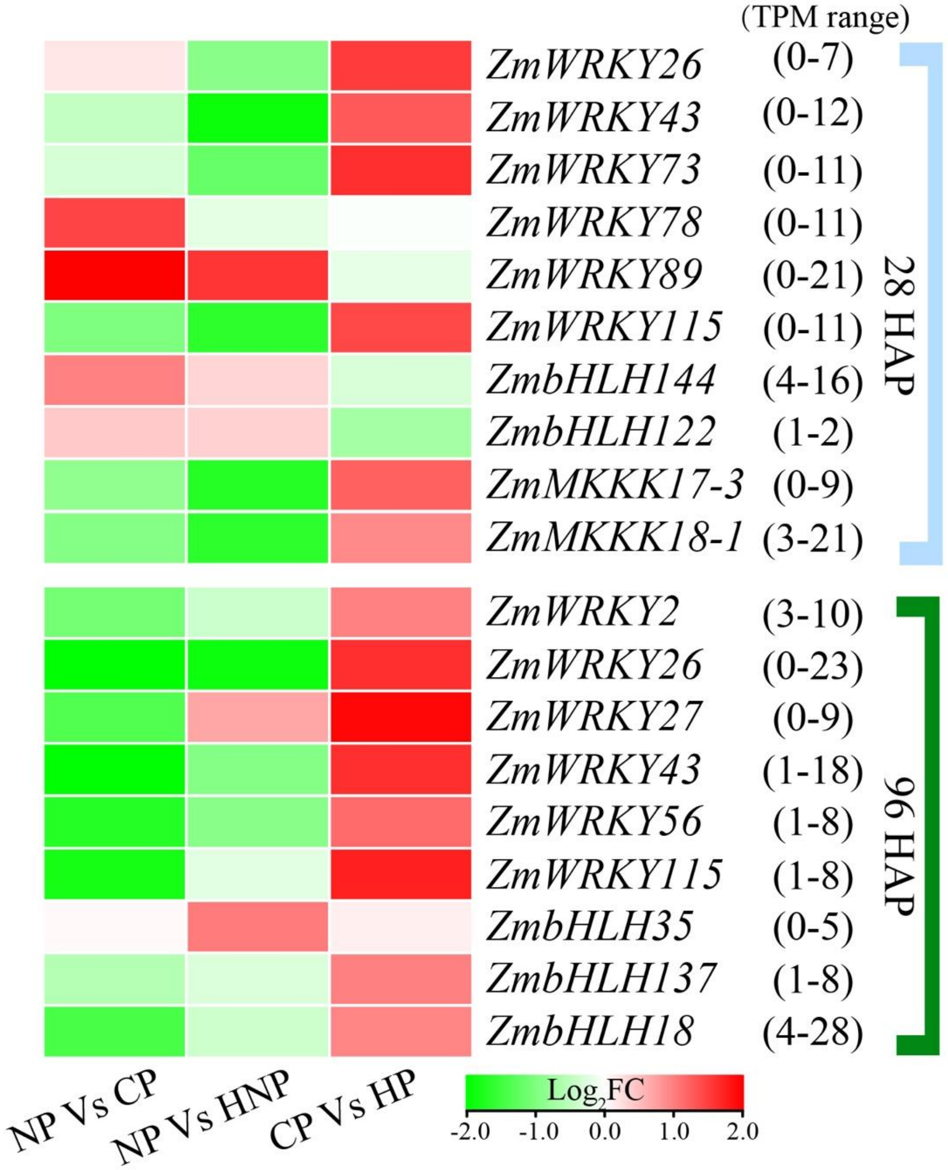

Supplementary Figure 5 Expression patterns of genes (with highest TPM less than 30) involved in MAPK signaling pathway at 28 and 96 HAP.

The MAPK signaling was largely suppressed in the HNP ovaries but promoted in the HP grains.

The absolute values of TPM in brackets represent the range between the minimum and maximum values of the gene in all treatments during that period. The fold change (FC) represents the ratio of the change in TPM value for each comparison. HAP, hours after pollination; NP, non-pollinated ovaries; CP, completely pollinated ovaries; HP, half pollinated ovaries; HNP, half non-pollinated ovaries; WRKY, WRKY-transcription factor; bHLH, basic helix-loop-helix transcription factor; MKKK, mitogen-activated protein kinase kinase kinase.

Supplementary Figure 6

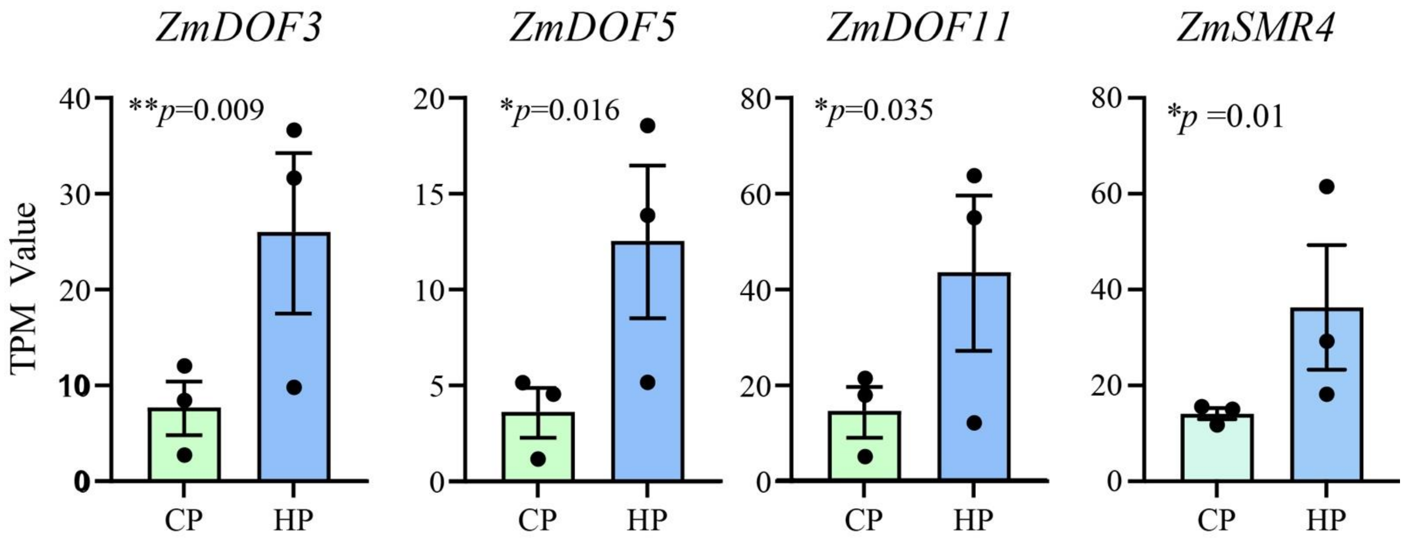

Supplementary Figure 6 Gene expression patterns of *ZmDOFs* and *ZmSMR4* at 96 HAP. *ZmDOFs*, transcription factors positively regulating sugar utilization and plant growth, and *ZmSMR4*, cell cycle-related gene, were promoted in the HP grains compared with that CP grains.

Conducted differential expression analysis on CP and HP using DESeq2 and obtained adjusted p-values to control for false discovery using the Benjamini-Hochberg method[1], *n*=3. CP, completely pollinated ovaries; HP, half pollinated ovaries.

Supplementary Figure 7

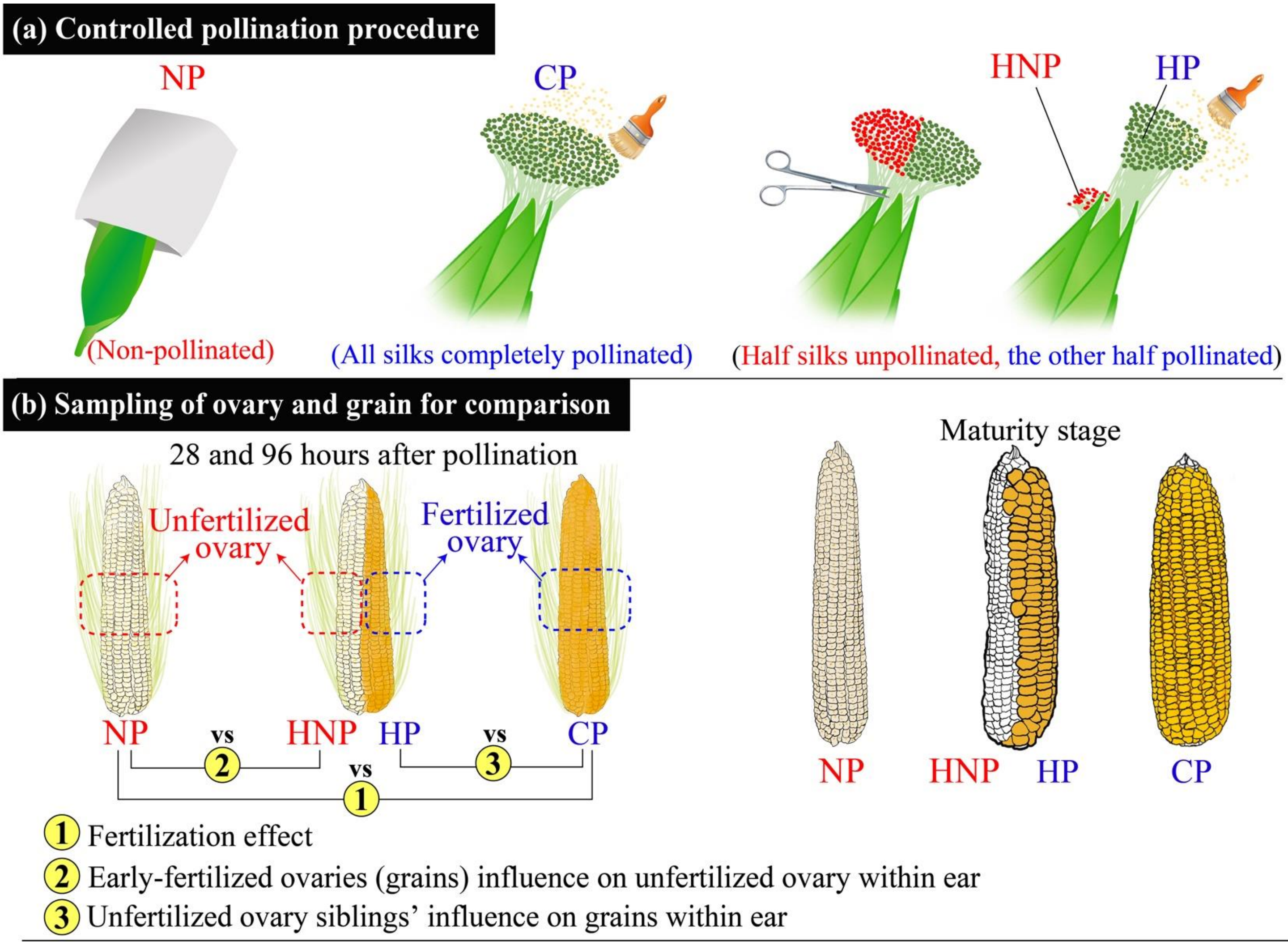

Supplementary Figure 7 Illustration demonstrating the treatments of manual pollination.

(a) Non-pollination (NP) and complete pollination (CP), where all silks were kept unpollinated and completely pollinated, were set as the negative and positive controls, respectively. In half pollination treatment, the silks from half rows of the ear were cut beneath the husks to prevent from pollination (HNP treatment), while pollinated the silks from another half rows with fresh pollens (HP treatment).

(b) At 28 and 96 hours after pollination, silks and ovaries from the middle region of ears were sampled for subsequent analyses. The impacts of interactions between grains and ovaries were obtained by the three pair comparisons. At maturity, grain phenotyping was conducted. The red and bule boxes indicate non-pollinated and pollinated ovaries or silks, respectively.

HAP, hours after pollination; NP, non-pollinated ovaries; CP, completely pollinated ovaries; HP, half pollinated ovaries; HNP, half non-pollinated ovaries.

Supplementary Figure 8

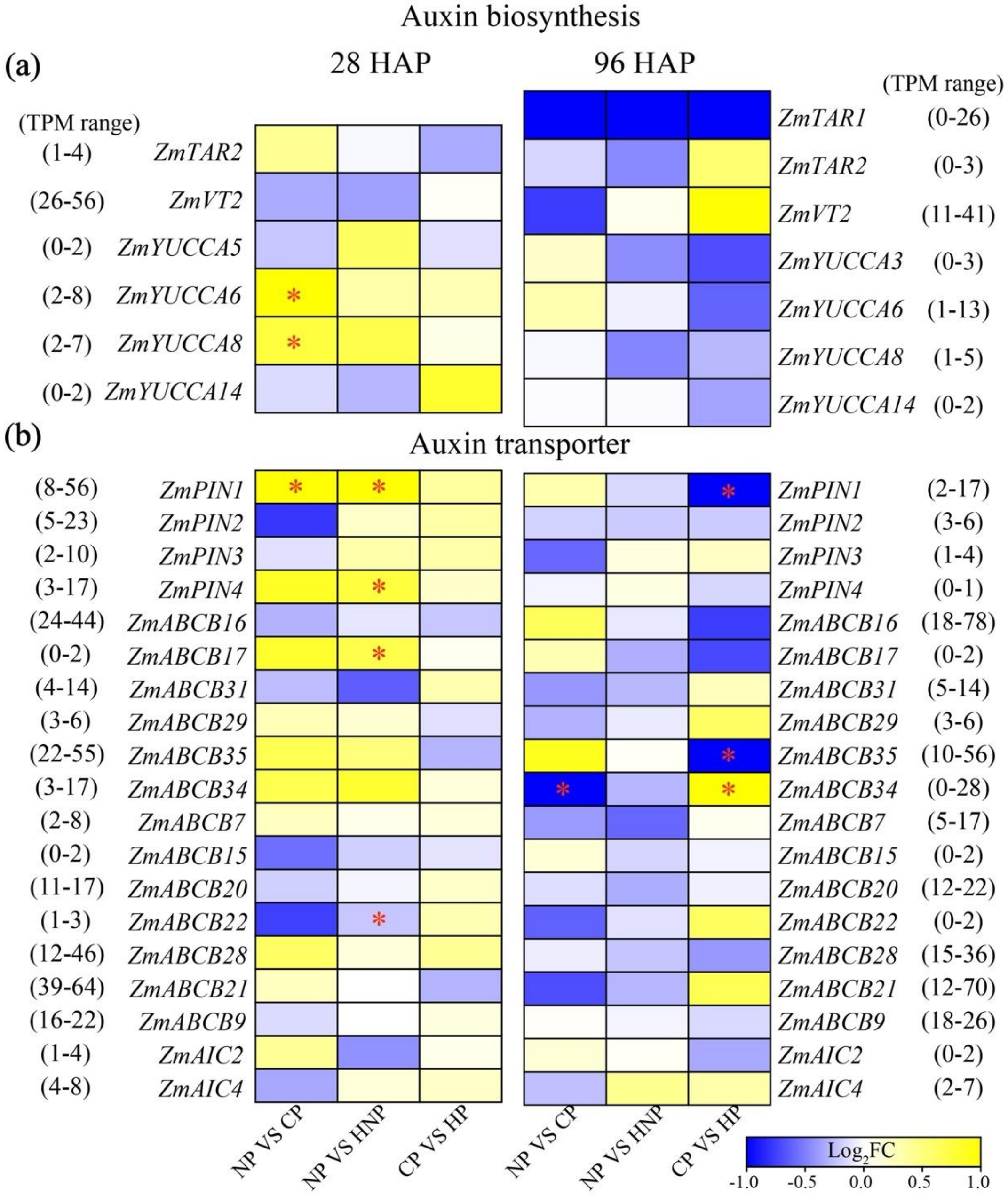

Supplementary Figure 8 Expression patterns of genes facilitating auxin biosynthesis and transport at 28 and 96 HAP.

(a) Genes facilitating auxin biosynthesis was promoted in the CP grains compared with NP ovaries, but no significant difference was observed for HNP and HP treatments.

(b) Several genes encoding auxin efflux and influx transporters, *ZmPIN1*, *4*, *ZmABCB17*, were significantly promoted in the HNP ovaries, but *ZmPIN1* and *ZmABCB35* were significantly reduced in the HP grains.

Red asterisk represents the significance of DEGs. The absolute values of TPM in brackets represent the range between the minimum and maximum values of the gene in all treatments during that period. The fold change (FC) represents the ratio of the change in TPM value for each comparison. HAP, hours after pollination; NP, non-pollinated ovaries; CP, completely pollinated ovaries; HP, half pollinated ovaries; HNP, half non-pollinated ovaries; TAR/VT, tryptophan aminotransferase; YUCCA, indole-3-pyruvate monooxygenase; PIN, PIN-formed protein (auxin efflux carrier); ABCB, ATP-binding cassette transporters (auxin influx carrier); AIC, auxin import carrier.

Supplementary Figure 9

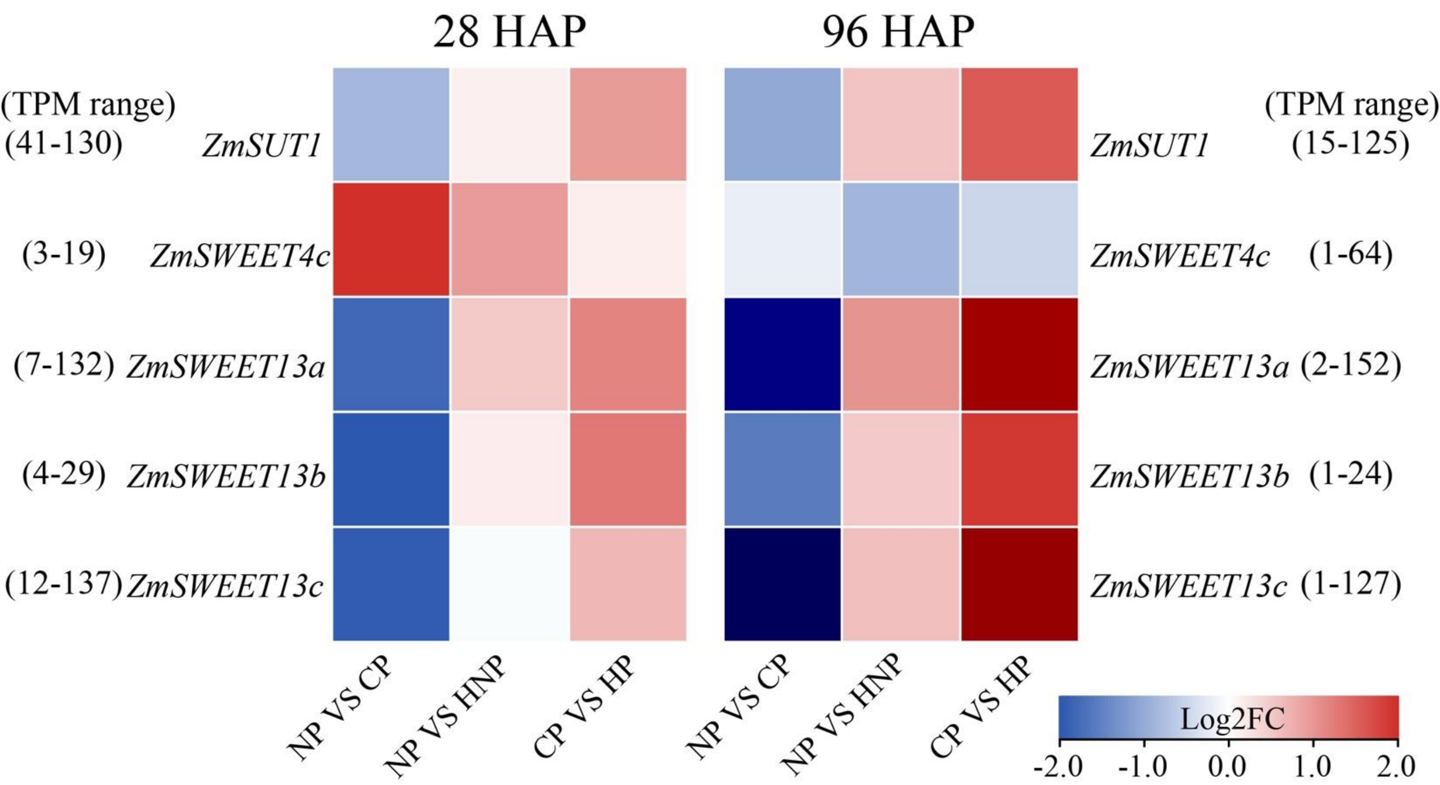

Supplementary Figure 9 Expression patterns of genes encoding sugar transporters at 28 and 96 HAP.

After fertilization, the majority of sugar transporters in the CP were downregulated compared to the NP. However, in both HNP and CP, genes encoding sugar transporters were upregulated compared to the NP and CP, respectively, at both time points. The absolute values of TPM in brackets represent the range between the minimum and maximum values of the gene in all treatments during that period. The fold change (FC) represents the ratio of the change in TPM value for each comparison. HAP, hours after pollination; NP, non-pollinated ovaries; CP, completely pollinated ovaries; HP, half pollinated ovaries; HNP, half non-pollinated ovaries; SUT, sucrose transporter; SWEET, sugars will eventually be exported transporter.

Supplementary Figure 10

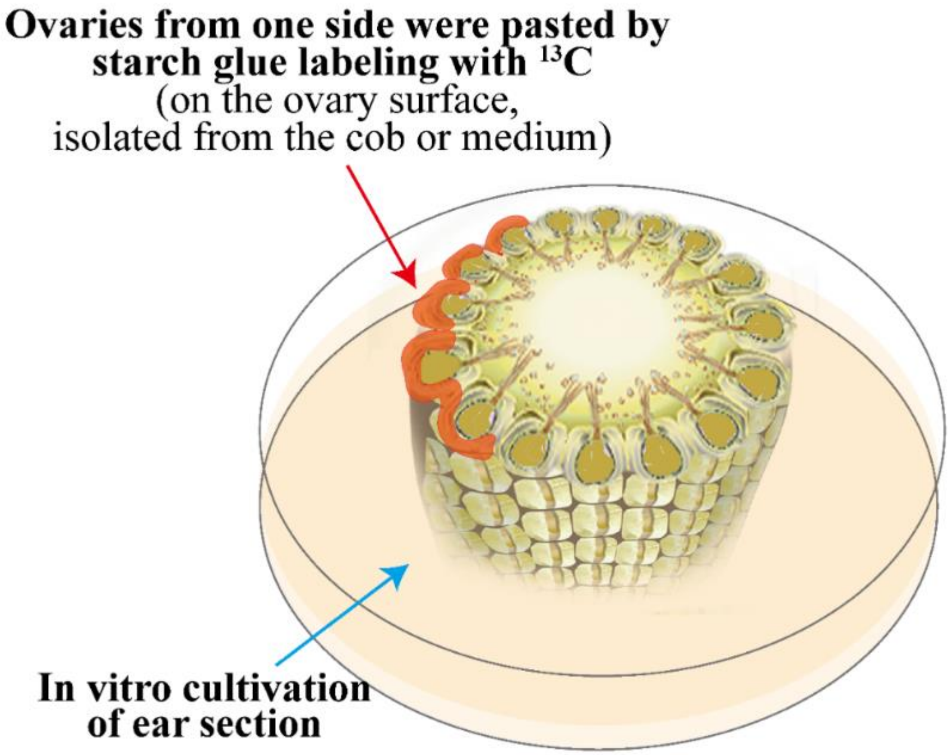

Supplementary Figure 10 Schematic diagram of i*n vitro* cultivation of ear section with ^{13}C-isotope labeling on one side of the ovaries/grains at 96 HAP.

^{13}C labelling of ovaries was achieved via sticking starch glue containing the ^{13}C isotope on the ovary without touching the medium or other ear tissues. After 5 days of incubation, the starch glue was washed off by distilled water, and the ^{13}C abundance of ovary/grain was detected using an elemental analyzer (elementar vario—PYRO cube, Germany) coupled to an isotope ratio mass spectrometer (isoprime100, UK).

Supplementary References

1. Love, M.I., Huber, W. and Anders, S. (2014) Moderated estimation of fold change and dispersion for RNA-seq data with DESeq2. Genome Biol, 15, 550.
