# Supplementary material for: Siblicide between fertilized and unfertilized ovaries within the maize ear
Source: Commun Biol. 2025 Mar 31;8:528. doi: 10.1038/s42003-025-07784-8 (PMC11958663; doi:10.1038/s42003-025-07784-8)
Supplement: Supplementary file 2 — Description of Supplementary Files [file 42003_2025_7784_MOESM2_ESM.pdf]

Description of Supplementary file

Description of Supplementary Data 1

Supplementary Data 1 provides the original data for all bar graphs.

Description of Supplementary Data 2

Supplementary Data 2 provides the original gene expression data for transcriptome.

Description of Supplementary Data 3

Supplementary Data 3 provides the original data for all heat map.
